# Variations in Microbial Diversity and Metabolite Profiles of Female Landrace Finishing Pigs With Distinct Feed Efficiency

**DOI:** 10.3389/fvets.2021.702931

**Published:** 2021-07-09

**Authors:** Zhixin Wang, Yingzhi He, Chuduan Wang, Hong Ao, Zhen Tan, Kai Xing

**Affiliations:** ^1^College of Animal Science and Technology, Hainan University, Haikou, China; ^2^Key Laboratory of Animal Genetics, Breeding, and Reproduction, Ministry of Agriculture, National Engineering Laboratory for Animal Breeding, College of Animal Science and Technology, China Agricultural University, Beijing, China; ^3^State Key Laboratory for Animal Nutrition, Key Laboratory for Domestic Animal Genetic Resources and Breeding of the Ministry of Agriculture of China, Institute of Animal Science, Chinese Academy of Agricultural Sciences, Beijing, China; ^4^Animal Science and Technology College, Beijing University of Agriculture, Beijing, China

**Keywords:** feed efficiency, microbial communities, metagenomics, metabolite, pigs

## Abstract

To enhance pig production, feed efficiency (FE) should be improved; however, the mechanisms by which gut microbes affect FE in pigs have not been fully elucidated. To investigate the differences between the composition and functionality of the gut microbiota associated with low and high FE, microbial compositions were characterized using 16S rRNA sequencing, functional annotations were performed by shotgun metagenomics, and metabolomic profiles were created by GC-TOF-MS from female Landrace finishing pigs with low and high feed conversion ratios (FCRs). *Lactobacillus* was enriched in the gut microbiota of individuals with low FCRs (and thus high FE), while *Prevotella* abundance was significantly higher in individuals with high FCRs (and thus low FE). This may be linked to carbohydrate consumption and incomplete digestion. The activity of pathways involved in the metabolism of cofactors and vitamins was greater in pigs with lower FE. We also identified differences in pyruvate-related metabolism, including phenylalanine and lysine metabolism. This suggests that pyruvate metabolism is closely related to microbial fermentation in the colon, which in turn affects glycolysis. This study deepens our understanding of how gut microbiota are related to pig growth traits, and how regulating microbial composition could aid in improving porcine FE. However, these results need to be validated using a larger pig cohort in the future.

## Introduction

Feed consumption is the largest variable expense associated with swine production, accounting for 50–85% of the total cost (1). Therefore, to enhance pig production, feed efficiency (FE) needs to be improved. Routine evaluation of FE is carried out using residual feed intake (RFI) or feed conversion ratio (FCR) values (2). A high FE means that an animal has a low RFI and FCR: gaining body weight while consuming less feed (2). Enhanced pig FE is associated with economic benefits in the swine industry, and progress has been made to optimize it using genetics, management practices, and dietary strategies (3).

The gut microbiota can ferment and metabolize nutrients such as, polysaccharides (to provide energy for the body), and regulate the energy harvest and carbohydrate metabolism of the host (4–6). Gut microbes are also significantly associated with weight gain of livestock (7–9). Although it does not necessarily have a major impact recent studies have reported possible links between the gut microbiota and pig FE (10), with some gut bacteria associated with and potentially serving as biomarkers for, desirable pig FE traits (11). These bacterial taxa could therefore be used as probiotics in dietary or breeding strategies to improve pig productivity. Many FE-associated bacteria are involved in carbohydrate degradation, including, those in the Christensenellaceae family and the *Treponema, Methanobrevibacter*, and *Actinobacillus* genera. These bacteria are enriched in the feces (12, 13), ileum (12, 14), caecum (15, 16) and colon (14) of pigs with high FE. By participating in polysaccharide degradation and subsequently producing a large amount of short-chain fatty acids, such as acetate, propionate, and butyrate, they provide an additional source of energy that can be directly utilized by the host *via* the intestinal tract. Some of the bacteria associated with improved FE are also capable of exerting anti-inflammatory effects, with some being particularly associated with butyrate production, including *Ruminococcus, Butyricicoccus, Roseburia*, and the Lachnospiraceae, which are enriched in feces and the ileum, caecum, and colon (11, 17). Some bacterial taxa linked to improved gut health and disease prevention are also FE-associated. For example, *Oscillibacter*, which was found to be more abundant in pigs with high FE, produces anti-inflammatory metabolites and is a useful probiotic (18). Additionally, *Lactobacillus* species are more prevalent in pigs with high FE and are commonly used as probiotics (15, 19). Most previous studies have focused on the association between the composition and predicted functions of the gut microbiota and FE. Gut microbiota metabolites are also closely related to host characteristics (20), with the regulation of host or microbe metabolites potentially altering the host phenotype (21–23). Host-microbiota interaction studies found that altered the diet composition could increase the animals intake of fructose and fructooligosaccharides and this was accompanied by the modulation of gut microbiota composition and functional pathways (especially for the degradation or biosynthesis of L-histidine), ultimately increased the production of short chain fatty acids (SCFAs) and promoted animals growth (24, 25).

Although many studies have been conducted on growth-related traits and gut microbiota, the results are sometimes conflicting. This may be due to differences in the studies animals and experimental conditions. However, a study that minimized such genetic, nutritional, and management diffienences also found multiple RFI-associated taxonomic differences, none of which were common to all geographic locations or batches within a location (13).

An increased understanding of the community structure and functional capacity of the gut microbiota will help elucidate the interaction between microbial activity and host physiology and metabolism. Therefore, in this study, colonic microbiota sequencing was undertaken to investigate the difference between the colonic digesta microbiota of two groups (high and low FE) of female finishing Landrace pigs, with regards to community structure and composition, functions, and metabolites. Correlations between these bacterial population variables and pig production performance were then identified. We hope that our founding will allow for a greater understanding of the microbial activity and material digestion that occurs in the large intestine, and how this affects the growth traits of pigs.

## Materials and Methods

### Animals and Sample Collection

In this study, we utilized 120 purebred female Landrace pigs, provided by Tianjin Ninghe Primary Pig Breeding Farm (Tianjin, China). All experimental pigs were weaned at the age of 28 days and were raised under similar feeding management regimes. When a pig's body weight reached 30 kg, it was transferred to the fattening room, where 10 pigs were housed in each pen. During the study, all experimental pigs were fed the same commercial formula diet and were kept under controlled farm management conditions. The feed was mainly composed of corn, soybean meal, lysine, and calcium hydrogen phosphate. This was available *ad libitum* from automated individual feeding systems (Velos; Nedap Co., Ltd., Groenlo, Netherlands), that recorded the feeding behaviors of each of the pigs, including the daily feed intake and daily body weight gain. All experimental pigs were healthy and antibiotic-free during the study period.

Two groups of 20 pigs were chosen for a further assessment based on feed and weight gain data obtained between 120 and 165 days: the HFCR group (low FCR and thus high FE) and the LFCR group (high FCR and thus low FE). The FCR values for these groups were significantly different (Supplementary Table 1). At 166 days, fresh fecal samples were collected from each animal's anus. Four pigs from each group with the most extreme FCR phenotypes were then selected and paired (two full-sibling pairs and two half-sibling pairs), with each pair having opposing FCR phenotypes (Supplementary Table 2). The selected pigs were euthanized and colonic digesta samples were immediately collected from each euthanized animal. After dipping the samples in liquid nitrogen, all samples were transferred to a freezer temperature −80°C until metagenomic analysis. These samples were allocated to one of two groups: the Lco group (colonic digesta samples from pigs with low FE and high FCR values) and the Hco group (colonic digesta samples from pigs with high FE and low FCR values).

All experimental procedures described in this study were approved by the Animal Welfare Committee of China Agricultural University (Permit Number: DK996) and conducted under the approved slaughtering guidelines (GB/T 17236–2008) of the Quality Supervision, Inspection, and Quarantine Committee of the People's Republic of China. All efforts were made to minimize animal suffering during the study.

### DNA Preparation and Sequencing Analysis

A QIAamp DNA Stool Mini Kit (Qiagen Ltd., Germany) was used to extract the fecal and colonic digesta DNA following the manufacturer's instructions. The V3–V4 region of the 16S rRNA gene was amplified (341F−806R) by polymerase chain reaction (PCR) using universal bacterial 16S rRNA gene PCR amplicon primers (26), forward primer was CCTAYGGGRBGCASCAG, the reverse primer was GGACTACNNGGGTATCTAAT. After purification, PCR products were used to construct the libraries. which were sequenced on an Illumina MiSeq platform, with 250 bp paired-end reads, and generated at Novogene (Beijing, China). The metagenomic sequencing of the eight colonic digesta DNA samples was performed using the Illumina HiSeq 2500 platform. The libraries were constrained according to Illumina's instructions, and bioinformatics analyses of the sequencing data were performed following the standard protocol (27).

The 16S rRNA gene sequence analyses were performed using QIIME (version 1.80) (28). Tags were clustered into operational taxonomic units (OTUs) at 97% similarity using UPARSE. Taxonomic assignments were screened against the 16S rRNA microbial reference database SILVA using the Ribosomal Database Project Classifier version 2.2 (29). Alpha and beta diversities were calculated using QIIME (30). The weighted pair-group method with arithmetic mean was used in R to cluster between-sample differences with evolutionary information. Linear discriminant analysis (LDA) effect size was used to identify biomarkers with significant differences in abundance among the different groups (31).

Clean short reads of the shotgun metagenomics were assembled using SOAPdenovo version 1.05 (32). Low quality reads, adaptor reads, and host sequences were removed. Redundant contigs were excluded to obtain maximum N50 values (27). The non-redundant contigs were mapped to microbial genomes in NCBI using SOAPdenovo (version 1.05). The aligned reads were classified at different microbial levels, and relative abundances were calculated. The assembled sequences were analyzed to predict open reading frames using MetaGeneMark (version 2.10). All predicted genes were clustered (identity > 95%, coverage > 90%) using CD-HIT (version 4.6.1). Clean reads were compared to the non-redundant gene set, which was constructed by removing redundant genes, using SOAPaligner and the parameters described above. The non-redundant gene set was mapped to the KEGG gene database to obtain KEGG ontology (KO) annotation information using BLAST (Version 2.2.28+). In addition, the non-redundant gene set was mapped to the Carbohydrate-Active Enzymes Database (CAZy) to acquire functional EC-classification information (33) for understanding metabolic mechanisms for the digestion of microbial carbohydrates. Differentially abundant genes were aligned to the Antibiotic-Resistance Genes Database (ARDB) to compare the types, quantities, and functions of the antibiotic-resistant (AR) genes in both groups (34). All sequencing data were deposited in the National Center for Biotechnology Information (NCBI) under the Sequence Read Archive (SRA) accession number SRP116179.

To identify differences in microbial communities and genes between the two groups, wilcox tests were carried out. The significance level was declared at *P* < 0.05 and adjusted by FDR (false discovery rate) with threshold value < 0.05. *Z*-score of row was calculated to homogenization control a heatmap to demonstrate the relative abundances of antibiotic-resistance genes between the colonic microbiota of the high- and low-FE groups.

### Untargeted Metabolomics Study and Data Analysis

Each colon content sample (50 mg) was transferred into a 2 mL tube, and 500 μL of a pre-cold extraction mixture of methanol/chloroform (3:1, v/v) with 10 μL of internal standard (L-2-Chlorophenylalanine, 1 mg/mL stock) was added. Next, the samples were vortexed for 30 s and homogenized with a ball mill for 4 min at 40 Hz, followed by ultrasonication for 5 min in ice water. This was repeated three times. After centrifugation (4°C, 12,000 rpm) for 15 min, 200 μL of the supernatant was transferred to a fresh tube. After evaporation in a vacuum concentrator, 50 μL of methoxyamination hydrochloride (20 mg/mL in pyridine) was added. The samples were then incubated at 80°C for 30 min, and then derivatized by 70 μL of BSTFA reagent (1% TMCS, v/v) at 70°C for 1.5 h. Gas chromatography coupled with a time-of-flight mass spectrometry analysis of the colonic contents was performed by Beijing Biomarker Technologies Co., Ltd. (Beijing, China) on an Agilent 7,890 gas chromatograph (Agilent, Germany). The raw data analysis, including peak extraction, baseline adjustment, deconvolution, alignment, and integration, was completed using Chroma TOF 4.3X software (LECO Corporation, St Joseph, MI, USA). The LECO-Fiehn Rtx5 database was used for metabolite identification by matching the mass spectrum and retention index (35).

## Results

### Sequencing, Assembly, and Taxon

**Figure 1:**
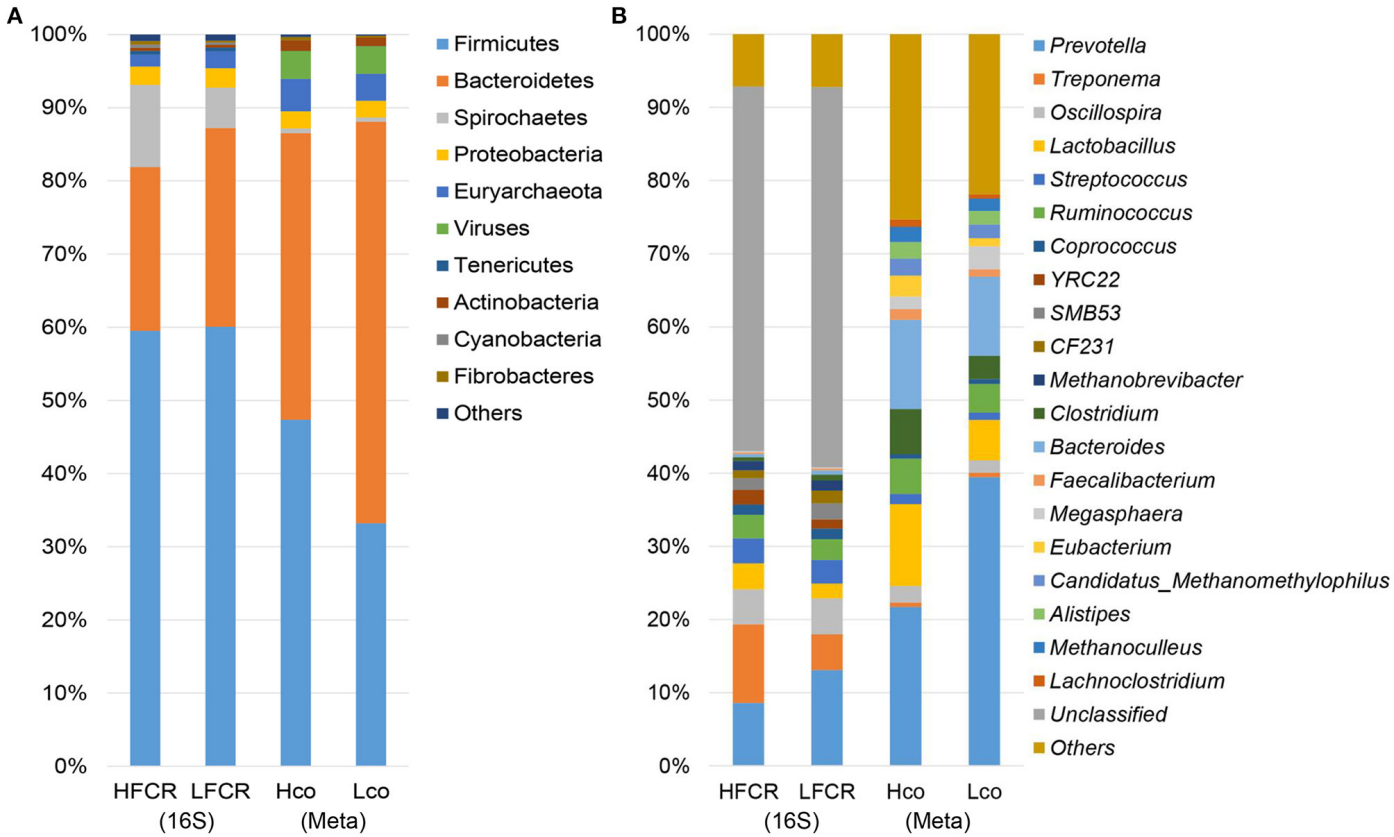
Histogram of bacteria at the **(A)** phylum and **(B)** genus level in multiple samples, based on fecal 16S rRNA gene and colonic shotgun metagenomic sequencing.

After quality control and demultiplexing, the number of available sequences for the 40 fecal samples ranged from 26,158 to 42,174. With a 97% identity cut-off as one OTU, the number of OTUs ranged from 1,658 to 3,030 (Supplementary Table 3). The bacterial diversity was compared between the HFCR and LFCR groups using diversity and richness estimators. The LFCR group had significantly higher Simpson, Shannon, PD_whole_tree, Chao1, and Observed_species indices than the HFCR group (*P* < 0.05; Supplementary Table 4). Firmicutes, Bacteroidetes, Spirochaetes, and Proteobacteria were the four most abundant phyla, accounting for more than 95% of the fecal sequences in both groups (Figure 1A). The most abundant sequences detected at the phylum level were from Firmicutes, comprising nearly 60% of all normalized reads; Bacteroidetes accounted for 22.36 and 27.16% of the notmalized reads in the HFCR and LFCR groups, respectively, while Spirochaetes accounted for 11.23 and 5.51%, respectively. Proteobacteria accounted for ~2.5% in both groups. At the genus level, a total of 293 genera were detected (Figure 1B), however, due to limitations of the targeted amplification sequencing, unclassified bacteria accounted for more than half of the total reads in both groups. Prevotella (HFCR, 7.97% abundance; LFCR, 12.23%), Treponema (HFCR, 10.74%; LFCR, 4.88%), Oscillospira (HFCR, 4.82%; LFCR, 4.90%), Streptococcus (HFCR, 3.43%; LFCR, 3.24%), Ruminococcus (HFCR, 3.03%; LFCR, 2.67%), and Lactobacillus (HFCR, 3.52%; LFCR, 2.01%) were the predominant genera detected.

Considering the limited resolution of the bacterial taxa based on the 16S rRNA gene sequencing data, eight colonic samples were taken from pigs with extreme FCR values (four samples from the HFCR group [Hco] and four from the LFCR group [Lco]. These samples were subjected to metagenomic sequencing analysis to identify the potential bacterial species associated with FE. A total of 1.2 million contigs with an average size of 1,094 and 1,161 bp with an average N50 length were obtained (Supplementary Table 5). Firmicutes and Bacteroidetes were the most abundant phyla as per *de novo* sequencing, this was a similar finding to that of the amplification sequencing (Figure 1A). At the genus level, contigs were assigned to a total of 1,271 genera, and the most abundant genera were Prevotella, Bacteroides, and Lactobacillus (Figure 1B). Across both the Hco and Lco groups, a total of 3,706 species of microbes were identified, but most had relatively low abundances. Prevotella, Bacteroides, Lactobacillus, Clostridium, and Ruminococcus were the five most abundant genera among the colonic microbes, representing more than half of the microbial population, on average.

### Association Between Microbial Composition and FE

Principal component analysis was used to compare the microbial composition of the feces from the HFCR and LFCR pig groups. Although some of the HFCR and LFCR samples had similar compositions, most could be clearly divided into two groups based on microbial abundance profiling by determining Bray–Curtis distances (Supplementary Figure 1). Using metagenomics, clear significant differences at both the genus and gene levels were observed between the colonic microbes of the Hco and Lco groups (Supplementary Figure 2). These differences indicate that the microbial community is significantly correlated with the FE of pigs.

**Figure 2:**
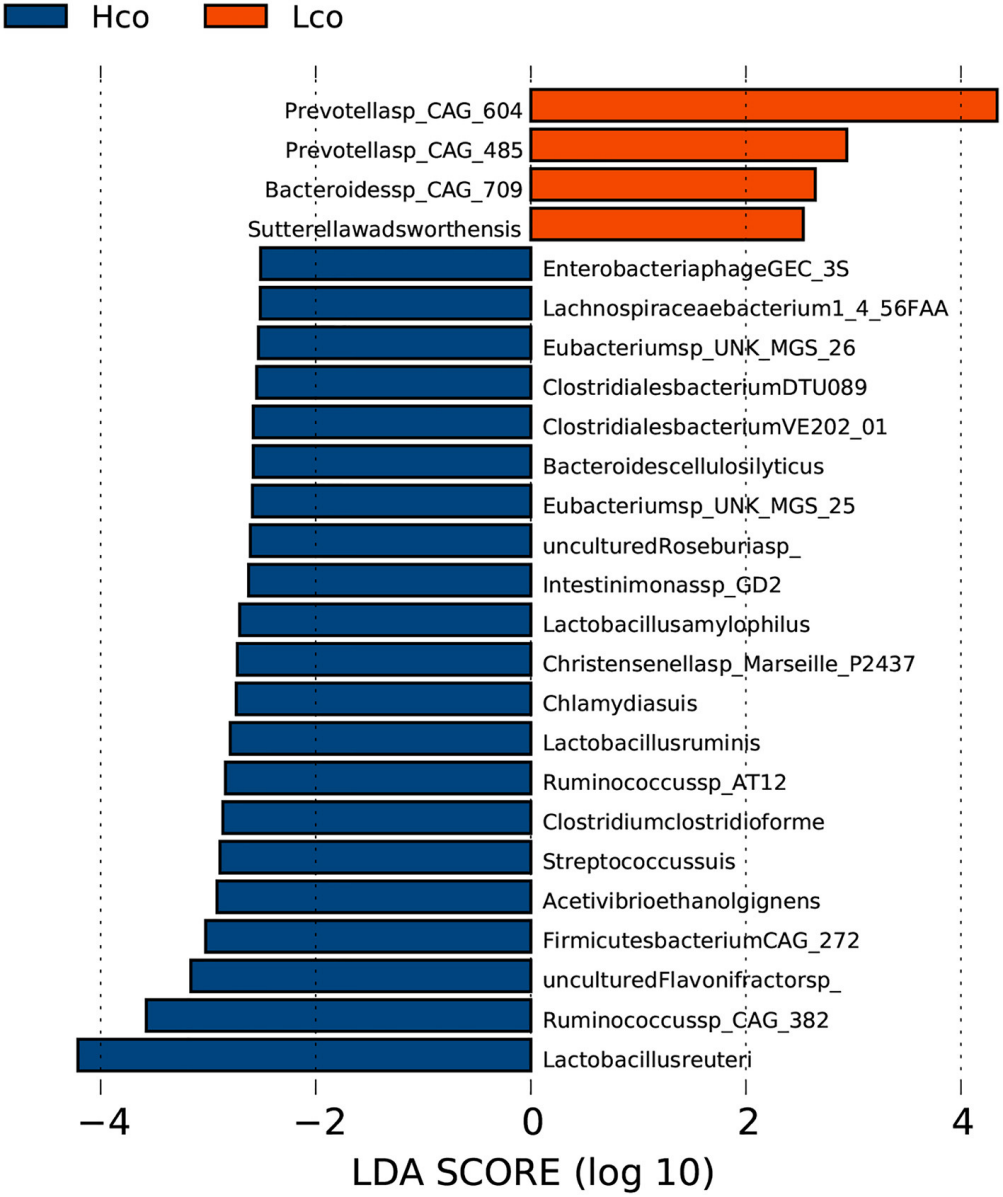
Identification of gut bacterial species associated with porcine FE using LEfSe analysis based on metagenomic sequencing data. Hco: colonic microbiota from pigs with high FE. Lco: colonic microbiota from pigs with low FE. The X-axis shows LDA scores. The LDA (linear-discriminant analysis) plot indicates biomarkers by ranking according to the effect size (2.0) for the species.

In the fecal samples, 25 genera were considered to be suitable biomarkers for distinguishing between high and low FE of finishing pigs (LDA score > 2). Eight genera were significantly enriched in the LFCR group, including Prevotella, while 17 genera were more abundant in the HFCR group, particularly Treponema and Lactobacillus (Supplementary Figure 3). A total of 177 out of 4,986 species showed significantly different relative abundances between the HFCR and LFCR groups, while some species were uniquely identified by LDA as biomarkers for colonic microbes in the Hco and Lco groups by LDA (Figure 2). Two species in particular were clearly distinguishable as potential biomarkers for FE, with LDA scores >4, one Prevotella species CAG: 604 was more abundant in the colonic digesta of the Lco group than in that of the Hco group, whereas Lactobacillus reuteri was more abundant in the digesta of Hco group.

### Comparison of Microbiome Functionality Between Hco and Lco Groups

**Figure 3:**
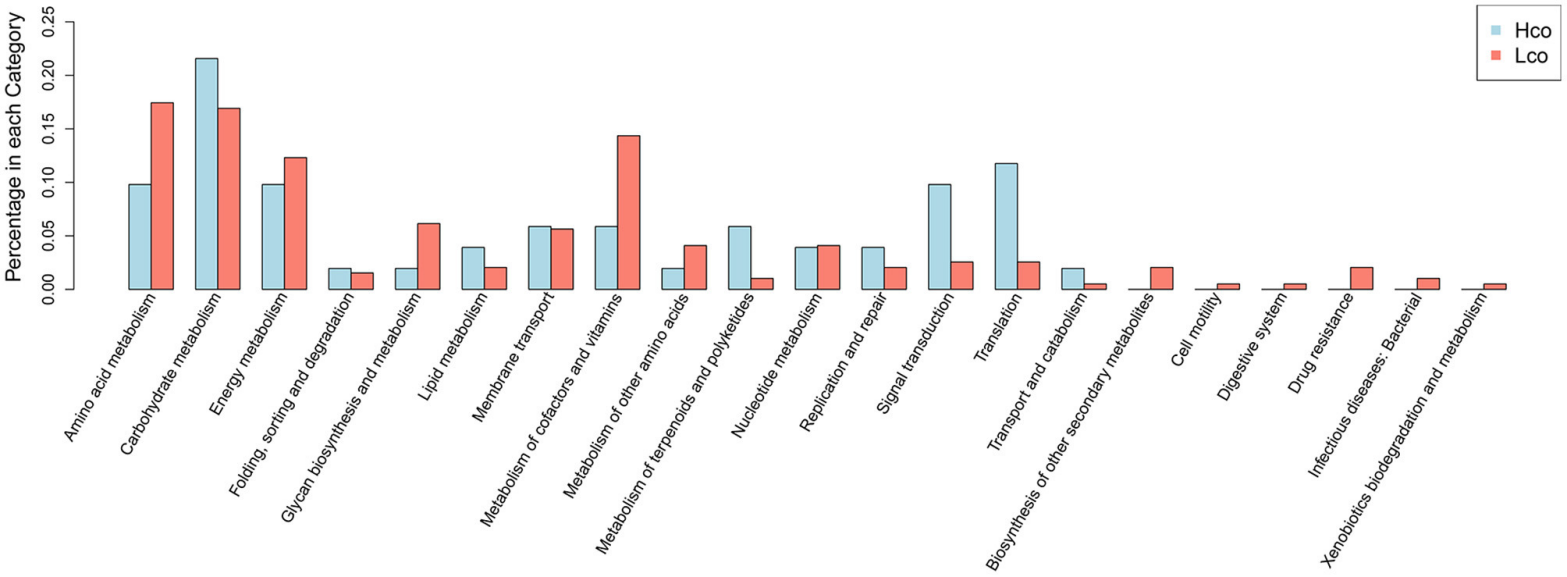
Distribution of differential KOs associated with porcine FE based on KEGG classification of metagenomic sequencing data. The abscissa indicates the name of the KEGG metabolic pathway and the ordinate indicates the number of KOs enriched for a certain function in the Hco or Lco group.

Differentially expressed colonic genes were annotated in the KEGG database (Supplementary Figure 4). Significantly different KOs between the Hco and Lco group samples were analyzed using the KO-annotation information obtained from the KEGG database, and the proportion of differential KOs in each classification was listed for both groups (Figure 3). Carbohydrate metabolism, signal transduction, and transcription were represented in relatively high proportions in the Hco group, whereas amino acid metabolism, energy metabolism, glycan biosynthesis and metabolism, and metabolism of cofactors and vitamins were more abundantly represented in the Lco group.

The CAZy database was used to align and categorize differentially abundant genes into seven CAZy types. In total, 776 and 1,541 genes from the Hco and Lco group, respectively, were mapped to the database. The proportions of CAZy types in the two groups were then compared (Supplementary Figure 5). Glycoside hydrolases (GHs) and glycosyl transferases (GTs) were more abundant in the Lco group samples than in the Hco group samples. The ARDB was used to annotate the abundance and strength of AR genes to identify coexisting microbes that produce antibiotics or toxins that compete for nutrients and inhibit the growth of other microbes in the colonic fluid. The abundance of AR genes in the samples was then presented in a heatmap after z-score processing (Supplementary Figure 6). The top five AR-type genes found in samples from both groups were “MacAB,” “aph,” “MLS_MFS,” “tet_efflux,” and “tet_RPP.” However, all AR genes clustered into two major functional subsets, and the subset distribution of the microbial-related AR genes in both groups differed. The growth of undesirable microbes in response to certain antibiotics may therefore be affected by the regulation of microbial composition.

Based on statistical analysis of the differential KOs, the differential pathways were investigated, and 22 pathways were found to be significantly different (*P* < 0.05, Table 1). Most of these pathways were metabolism-related, including amino acid metabolism, carbohydrate metabolism, energy metabolism, glycan biosynthesis and metabolism, nucleotide metabolism and metabolism of cofactors, and vitamins.

**Table 1:** Different KO pathways observed for porcine colonic microbiota in the Hco and Lco groups.

**KO pathway**	**Description**	**Hco**	**Lco**	***P*-value**
Alanine, aspartate and glutamate metabolism	Amino acid metabolism	0.0098	0.0109	0.0286
Phenylalanine, tyrosine and tryptophan biosynthesis	Amino acid metabolism	0.0052	0.0058	0.0286
Lysine biosynthesis	Amino acid metabolism	0.0045	0.0049	0.0286
Citrate cycle (TCA cycle)	Carbohydrate metabolism	0.0050	0.0055	0.0286
Amino sugar and nucleotide sugar metabolism	Carbohydrate metabolism	0.0092	0.0098	0.0286
Carbon fixation pathways in prokaryotes	Energy metabolism	0.0090	0.0097	0.0286
Nitrogen metabolism	Energy metabolism	0.0034	0.0037	0.0286
Lipopolysaccharide biosynthesis	Glycan biosynthesis and metabolism	0.0022	0.0028	0.0286
Peptidoglycan biosynthesis	Glycan biosynthesis and metabolism	0.0060	0.0064	0.0286
Thiamine metabolism	Metabolism of cofactors and vitamins	0.0028	0.0032	0.0286
Ubiquinone and other terpenoid-quinone biosynthesis	Metabolism of cofactors and vitamins	0.0008	0.0011	0.0286
One carbon pool by folate	Metabolism of cofactors and vitamins	0.0043	0.0046	0.0286
Vitamin B6 metabolism	Metabolism of cofactors and vitamins	0.0016	0.0017	0.0286
Porphyrin and chlorophyll metabolism	Metabolism of cofactors and vitamins	0.0029	0.0033	0.0286
Riboflavin metabolism	Metabolism of cofactors and vitamins	0.0010	0.0012	0.0286
Pantothenate and CoA biosynthesis	Metabolism of cofactors and vitamins	0.0035	0.0040	0.0286
Nicotinate and nicotinamide metabolism	Metabolism of cofactors and vitamins	0.0031	0.0035	0.0286
Taurine and hypotaurine metabolism	Metabolism of other amino acids	0.0010	0.0011	0.0286
Terpenoid backbone biosynthesis	Metabolism of terpenoids and polyketides	0.0036	0.0039	0.0286
Purine metabolism	Nucleotide metabolism	0.0191	0.0202	0.0286
Biosynthesis of amino acids	Overview of metabolism	0.0314	0.0340	0.0286
Homologous recombination	Replication and repair in Genetic information processing	0.0083	0.0086	0.0286

### Metabolite Profiles in the Colonic Digesta of Hco and Lco Groups

A total of 429 metabolite characteristics of the colonic digesta were detected using GC-TOF-MS. To distinguish the differences between Hco and Lco group samples, orthogonal projections to latent structures-discriminant analysis (OPLS-DA) was performed using the R (version 3.3.2) package ropls. The values of R2Y and Q2Y were 0.998 and 0.571, respectively, indicating that the model built by the OPLS-DA method could distinguish the correct sample group by metabolic expression; it could therefore be used to screen for differential metabolites (Supplementary Figure 7). At the significance threshold of fold change value >1, *P*-value < 0.05, and variable importance in projection value > 1, nine metabolites were detected with significant associations between microbial metabolites and FE (Table 2). Among these nine metabolites, six had a tendency to be negatively correlated with FCR, including tetracosane, palmitoleic acid, linolenic acid, and 2-Indanone, while three metabolites showed a tendency to be positively correlated with FCR, including 3-(3-hydroxyphenyl) propionic acid. The pathway analysis showed that metabolites were enriched in 60 metabolic pathways, including microbial metabolism in diverse environments, biosynthesis of secondary metabolites, biosynthesis of plant secondary metabolites, and phenylalanine metabolism. Four differential metabolites were enriched in KEGG pathways (Table 2), palmitoleic acid was related to fatty acid biosynthesis, while the other three metabolites were related to multiple metabolic pathways.

**Table 2:** Summary of differential metabolites and their functional KEGG annotations associated with different porcine feed efficiencies.

**ID**	**MetaboliteNames**	**HC_Mean**	**LC_Mean**	**Fold_change**	***P*-value**	**VIP**	**Regulated**	**KEGG_pathway_annotation**
meta_129	Tetracosane	0.014	0.035	2.442	0.047	1.852	Up	–
meta_160	Palmitoleic acid	0.020	0.034	1.636	0.028	2.067	Up	Fatty acid biosynthesis
meta_201	Linolenic acid	0.141	0.215	1.521	0.022	1.996	Up	Alpha-Linolenic acid metabolism; Biosynthesis of secondary metabolites; Metabolic pathways; Biosynthesis of plant secondary metabolites; Biosynthesis of unsaturated fatty acids; Biosynthesis of plant hormones
meta_310	Analyte 64	0.003	0.005	1.653	0.037	2.071	Up	–
meta_328	Analyte 593	0.080	0.032	0.395	0.042	1.963	Down	–
meta_333	Analyte 584	0.005	0.002	0.363	0.042	1.973	Down	–
meta_342	Analyte 56	0.001	0.002	2.150	0.030	2.041	Up	–
meta_553	3-(3-hydroxyphenyl) propionic acid	0.019	0.010	0.510	0.050	1.931	Down	Phenylalanine metabolism; Degradation of aromatic compounds; Microbial metabolism in diverse environments
meta_559	2-Indanone	0.005	0.010	1.925	0.029	1.976	Up	Microbial metabolism in diverse environments; Polycyclic aromatic hydrocarbon degradation

## Discussion

Recently, with the widespread application of next-generation sequencing, the understanding of the mechanisms by which microbes affect their hosts has considerably increased. Studies have found that approximately one-tenth of the host transcriptome is regulated by microorganisms (36). Any microorganism changes may therefore cause changes to the host phenotype. Many studies, including our previous study, have confirmed that there are differences in the microbial structure of pigs with different FEs (12, 16, 37–39). Feed efficient pigs showed superior antioxidant, metabolic, and cell repair capabilities in the mitochondria of multiple tissues compared to pigs with low FE (37–40). The body adjusts the composition of the gut microbiota composition and fermentation products by regulating digestion and secretion of the fermentation substrates of the microorganisms in various gut regions (11). This is an important factor for FE-related microbial community in the large intestine. There are significant differences in the proportions of certain bacteria in the guts of pigs with different FEs. For example, Oscilibacter, Christensenellaceae, and Cellulosilyticum are enriched in high-FE pigs (12). Ruminococcaceae, Christensenellaceae, Akkermansia, and Lachnospiraceae are also reported to be more abundant in high-FE pigs, whereas Faecalibacterium has a negative association with porcine FE (41). Studies have shown that differences in the composition and function of the gut microbiota can lead to physiological and functional changes related to the pig's FE and growth. However, the results of these studies differed (11), therefore, further research is necessary.

The results of our 16S sequencing showed that pigs with lower FE had greater alpha diversity in their gut microbiota than pigs with higher FE, which is not consistent with previous studies (11); however, diversity was also inconsistent within the study groups. This might be because some samples had similar microbial composition despite being in different groups; the study was also likely affected by the limited sample size. Regardless of FE, the overall distribution of dominant bacteria was consistent with other studies (8). The bacterial genera *Prevotella, Treponema*, and *Lactobacillus* were also found in other studies, and our metagenomic results found that the two most significantly different species in terms of their abundance in each group were *Prevotella* sp. CAG_604 and *Lactobacillus reuteri*. Gene annotation showed that many genes in both groups belonged to the *Oscillospira* genus, the growth of which is probably induced by potentially pathogenic bacteria and can be considered a sign of a healthy gut (42). In addition, in the Hco group, most genes belonged to *Bacteroides* and *Lactobacillus*. Contrastingly, in the Lco group, most genes were annotated to *Prevotella*. These results are consistent with previously identified differences between high and low FE groups. In our previous studies, differentially expressed genes in the cecal and colonic mucosa of animals with high and low FE were mostly related to immunity and disease (43). The results of the cecal metagenome revealed that pyruvate-related metabolic pathways are significantly different between high- and low-FE groups (15). In this study, data from the colonic metagenomic and metabolome analyses also revealed differences in pyruvate-related metabolism, including phenylalanine and lysine metabolism. This suggests that pyruvate metabolism is closely related to microbial fermentation in the large intestine, which in turn affects glycolysis. A higher number of CAZy enzymes (CAZymes) is associated with better digestive capacity in pigs (44). The identification of CAZymes in the assembled set of differentially abundant genes suggests that the variable microbiome evident in this study may have formed due to microbial interactions with the surrounding environment, especially owing to available nutrients. The number of differentially enriched genes varied between groups, and more genes were clearly mapped to the database in the Lco group than in the Hco group. The distribution of genes encoding six enzymes showed similar distributions. Predominant enzymes were GHs and GTs, but differences in distributions were noted for each type.

The large number of coexisting microbes in the gut lumen causes competition for nutrients, and certain microbes may secrete antibiotics or bacteriocins to inhibit the growth of other bacterial species. The expression level and strength of antibiotics in individuals can be annotated using ARDB (34). The annotated AR genes in the individual pigs of each group (Hco and Lco) were divided into two categories on a heatmap. The AR genes in both categories also differed in the cluster. The diversity of growth was correlated with antibiotic level; thus, targeting and regulating AR genes may be helpful for distinguishing species and promoting the healthy growth of hosts.

All 22 upregulated pathways belonged to the Lco group due to the higher number of differentially abundant genes (Table 1). Eight different pathways were related to the metabolism of cofactors and vitamins; the colon itself does not perform digestion, but microbes in the colon can digest cellulose and synthesize vitamins. The different metabolic pathways were significantly enriched in the Lco group partly because of the larger number of genes with significant expression differences in this group. This is potentially due to the incomplete digestion of colonic nutrients, leaving more food residue in the colon, and thus leading to greater microbial activity. Our findings might be partially explained by the small sample size, which was difficult to adjust for effectively, and may have caused a certain degree of false-positive results. Therefore, the results of the study should be verified with a larger sample size in future trials.

## Conclusion

In summary, the various fecal and colonic microbiota of finishing pigs were correlated with different FEs. For example, *Lactobacillus* tended to be enriched in pigs with high FE. The abundance of *Prevotella* found in pigs with low FE may be linked to the consumption of carbohydrates that were incompletely digested. Our functional analysis suggests that the proportion of differentially abundant genes affects host metabolism. The pathways mediating the metabolism of cofactors and vitamins were significantly different between groups. Furthermore, related genes were linked to different microbes in the two groups. Data from the colonic metagenomic and metabolome analyses also revealed differences in pyruvate-related metabolism, including phenylalanine and lysine metabolism. This suggests that pyruvate metabolism is closely related to microbial fermentation in the large intestine, which in turn affects glycolysis. We have shown that genomics-sequencing technique is convenient for the study of pig gut microbial community structure, function, and host gene expression, and our preliminary results provide a starting point and reference for subsequent testing, thus enhancing the understanding of the interaction between pig FE and gut microbes.

## Data Availability Statement

The sequencing data were deposited in the National Center for Biotechnology Information (NCBI) under the Sequence Read Archive (SRA) Accession Number SRP116179.

## Ethics Statement

The animal study was reviewed and all experimental procedures described in this study were approved by the Animal Welfare Committee of China Agricultural University (Permit Number: DK996) and con-ducted under the approved slaughtering guidelines (GB/T 17236–2008) from the Quality Supervision, Inspection, and Quarantine Committee of the People's Republic of China.

## Author Contributions

ZT and CW: conceptualization and funding acquisition. ZW, KX, and ZT: methodology and software. ZW, YH, and HA: resources. ZW, YH, KX, and ZT: data curation. ZW and ZT: writing-original draft preparation and visualization. ZT and KX: writing-review and editing. KX and CW: project administration. All authors have read and agreed to the published version of the manuscript.

## Conflict of Interest

The authors declare that the research was conducted in the absence of any commercial or financial relationships that could be construed as a potential conflict of interest.

## Supplementary Material

Supplementary Figure 1 Principal component analysis (PCA) of fecal microbes in pigs with high and low feed conversion ratios (FCRs). The result shows the distinct composition of the fecal microbiome in these two groups.

Supplementary Figure 2 Different bacterial compositions at the genus and gene levels in colonic digesta samples from pigs with high (Hco) and low (Lco) FE. **(A)** PCA of metagenomics genera. **(B)** PCA of predicted genes. Differences between boxes were tested by the Wilcoxon test (^*^*P* < 0.05).

Supplementary Figure 3 Identification of fecal bacterial genera associated with porcine FE using the LEfSe analysis based on 16S rRNA gene sequencing data. The X-axis shows LDA scores. The LDA (linear-discriminant analysis) plot indicates biomarkers by ranking according to the effect size (2.0) for the species.

Supplementary Figure 4 Classification statistics of KEGG-annotation results for the Hco and Lco groups. The ordinate is the name of the level-1 KEGG metabolic pathway, and the abscissa is the number of genes annotated to the pathway.

Supplementary Figure 5 Distribution of differentially abundant genes clustered by CAZy in the Hco and Lco groups. CAZy, Carbohydrate-Active Enzymes Database; Hco, the proportion of significantly enriched genes in the samples from four pigs with the highest FE; Lco, the proportion of significantly enriched genes in the samples from four pigs with the lowest FE. The abscissa represents the CAZy classification. The ordinate represents the proportion of differentially expressed genes for each enzyme classification.

Supplementary Figure 6 Heatmap diagram showing differences in the abundances of antibiotic resistant genes in the colonic microbiota between the Hco (high FE) and Lco (low FE) groups. FE, feed efficiency. Homogenization control of rows is by z-score. Rows represent the types of antibiotic-resistant genes, and the columns represent the samples.

Supplementary Figure 7 OPLS-DA of the microbial metabolites in pigs with high and low feed conversion ratios (FCRs). OPLS-DA, orthogonal projections to latent structures- discriminant analysis. R2X and R2Y represent the interpretation rate of the built model to the X and Y matrices, respectively, where the X matrix is the metabolite quantitative matrix, the Y matrix is the sample-grouping matrix, and Q2 is the predictive ability if the model can distinguish the correct sample grouping by metabolic expression.

Supplementary Table 1 Feed conversion ratios (FCRs) for fecal samples of selected individuals. DFI, daily feed intake; ADG, average daily gain over the assessed feeding period; BW, body weight.

Supplementary Table 2 Individuals selected for metagenomics analysis. Half-siblings, H1co and L1co, H2co and L2co; Full-siblings, H3co and L3co, H4co and L4co.

Supplementary Table 3 16S rRNA gene amplicon sequencing of fecal microbes from pigs with high and low feed efficiency (FE).

Supplementary Table 4 Alpha diversity indices of fecal microbes in pigs with high and low feed efficiency (FE). *n* = 20 in each measurement. Shannon and Simpson indices were used to assess biodiversity. PD whole tree index was based on the phylogenetic tree. Chao1 indices were used to estimate the number of OTUs and microbial richness. The observed species index shows the number of OTUs actually observed.

Supplementary Table 5 Summary of metagenomic sequencing data.
