# Supplementary figures and images for: Variations in Microbial Diversity and Metabolite Profiles of Female Landrace Finishing Pigs With Distinct Feed Efficiency
Source: Front Vet Sci. 2021 Jul 9;8:702931. doi: 10.3389/fvets.2021.702931 (PMC8299115; doi:10.3389/fvets.2021.702931)

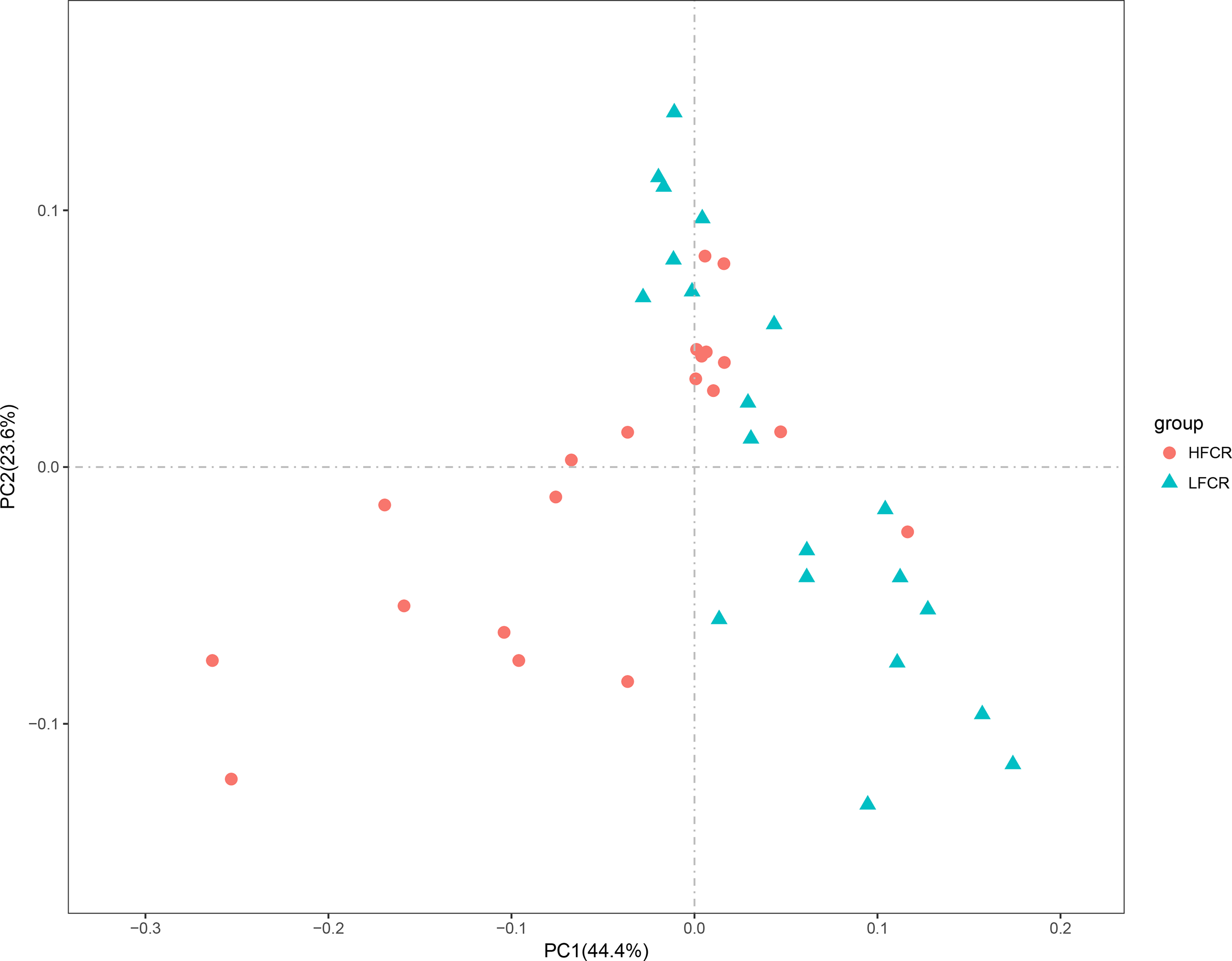

Supplement: Supplementary Figure 1 — Principal component analysis (PCA) of fecal microbes in pigs with high and low feed conversion ratios (FCRs). The result shows the distinct composition of the fecal microbiome in these two groups. [file Image_1.TIF]

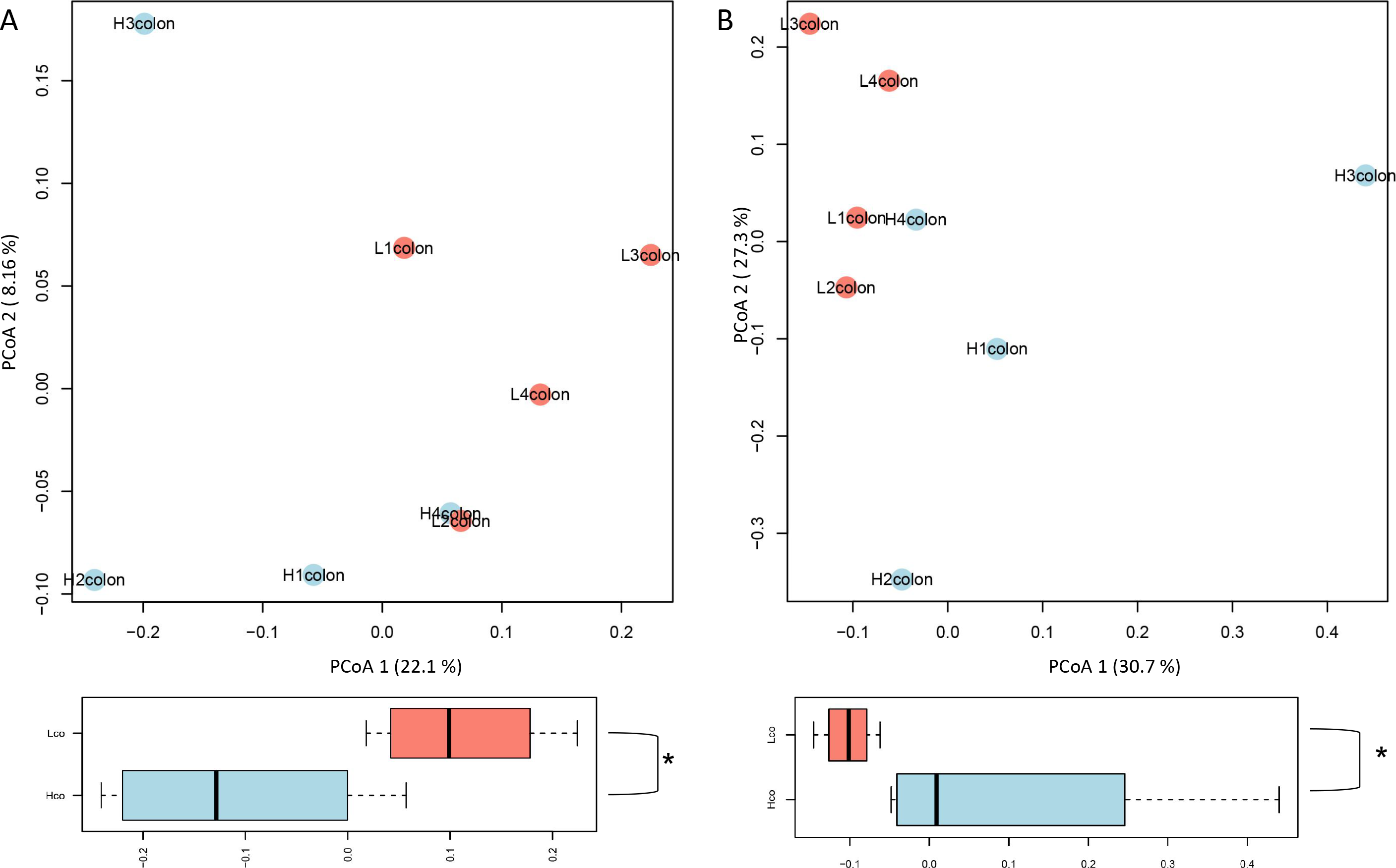

Supplement: Supplementary Figure 2 — Different bacterial compositions at the genus and gene levels in colonic digesta samples from pigs with high (Hco) and low (Lco) FE. (A) PCA of metagenomics genera. (B) PCA of predicted genes. Differences between boxes were tested by the Wilcoxon test (*P < 0.05). [file Image_2.TIF]

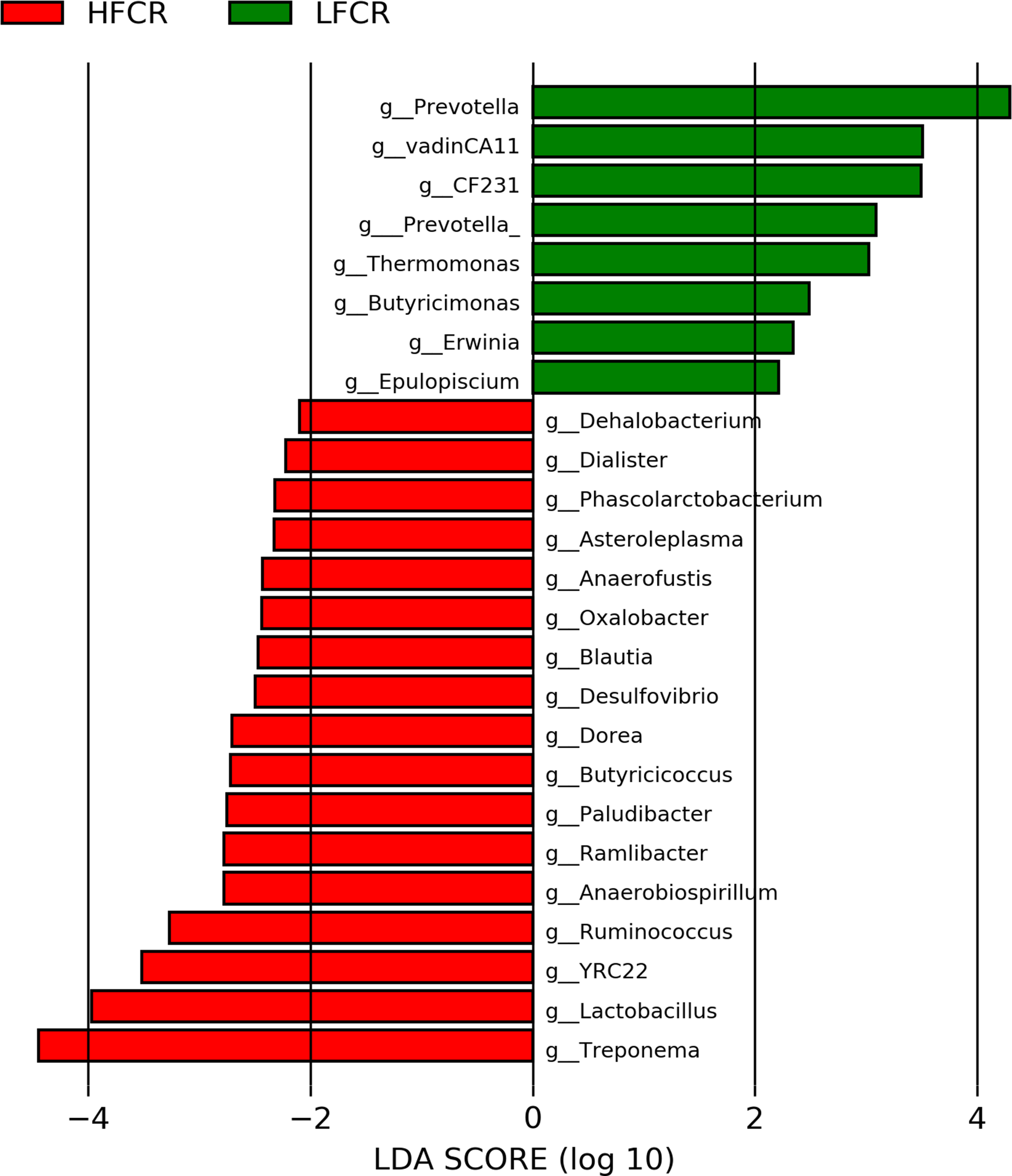

Supplement: Supplementary Figure 3 — Identification of fecal bacterial genera associated with porcine FE using the LEfSe analysis based on 16S rRNA gene sequencing data. The X-axis shows LDA scores. The LDA (linear-discriminant analysis) plot indicates biomarkers by ranking according to the effect size (2.0) for the species. [file Image_3.TIF]

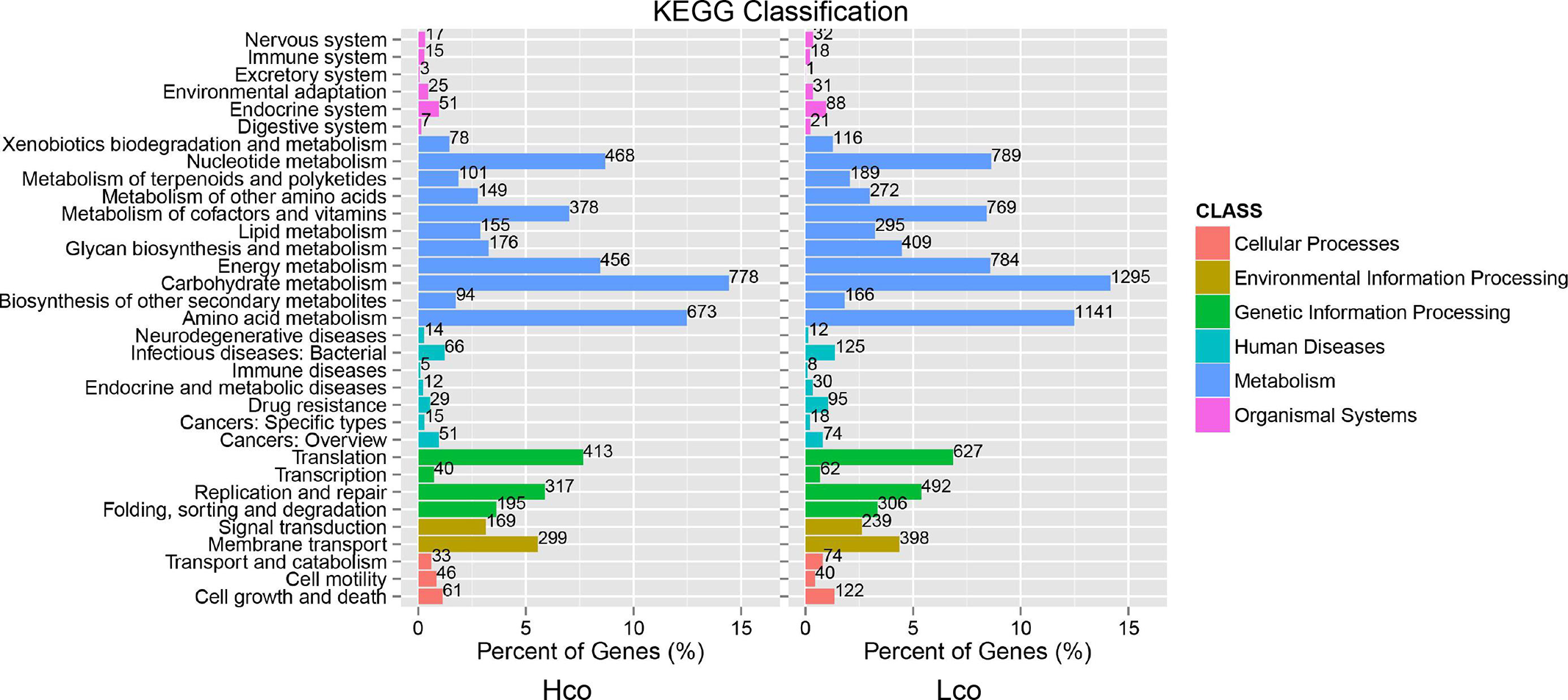

Supplement: Supplementary Figure 4 — Classification statistics of KEGG-annotation results for the Hco and Lco groups. The ordinate is the name of the level-1 KEGG metabolic pathway, and the abscissa is the number of genes annotated to the pathway. [file Image_4.TIF]

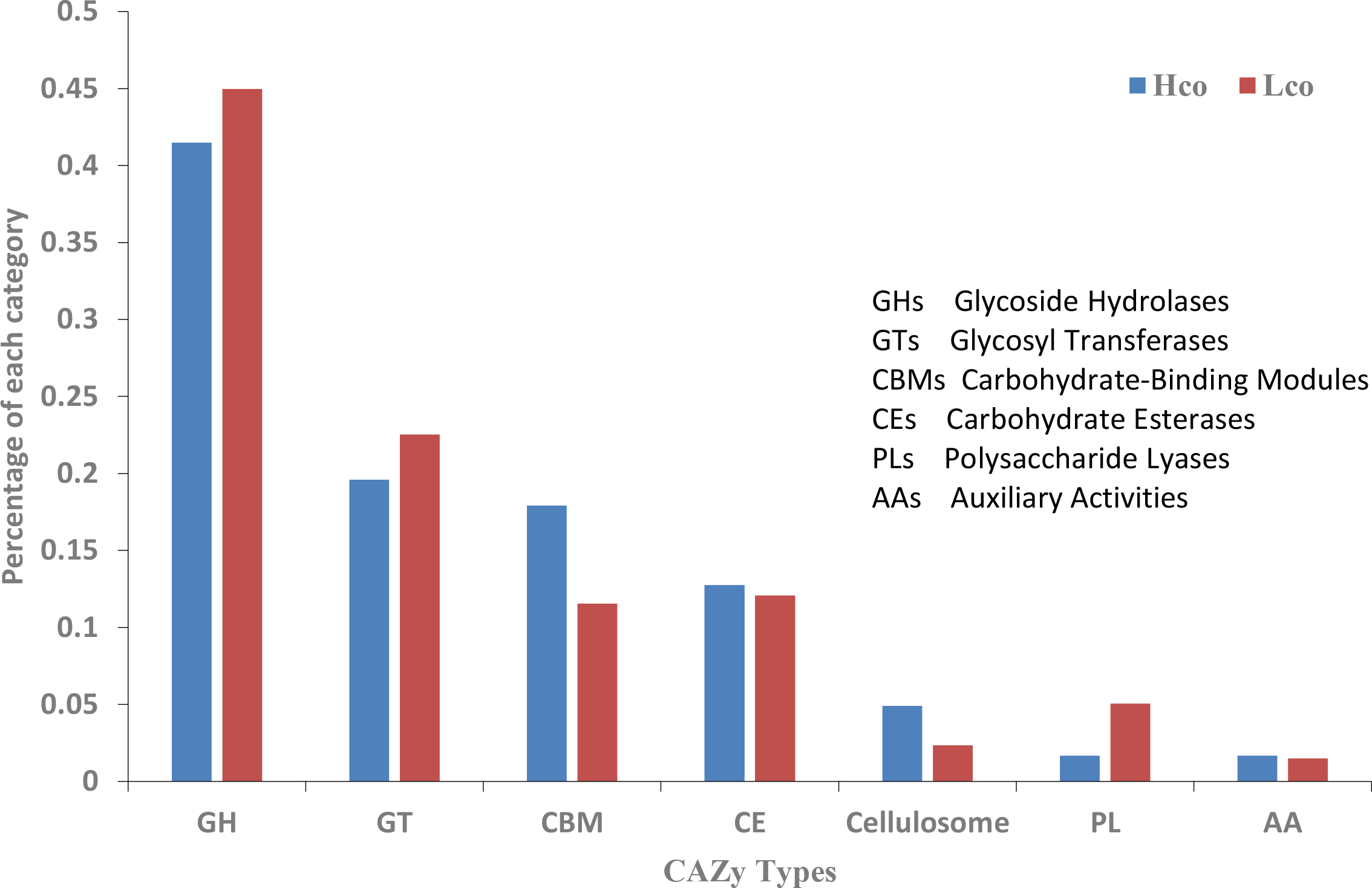

Supplement: Supplementary Figure 5 — Distribution of differentially abundant genes clustered by CAZy in the Hco and Lco groups. CAZy, Carbohydrate-Active Enzymes Database; Hco, the proportion of significantly enriched genes in the samples from four pigs with the highest FE; Lco, the proportion of significantly enriched genes in the samples from four pigs with the lowest FE. The abscissa represents the CAZy classification. The ordinate represents the proportion of differentially expressed genes for each enzyme classification. [file Image_5.TIF]

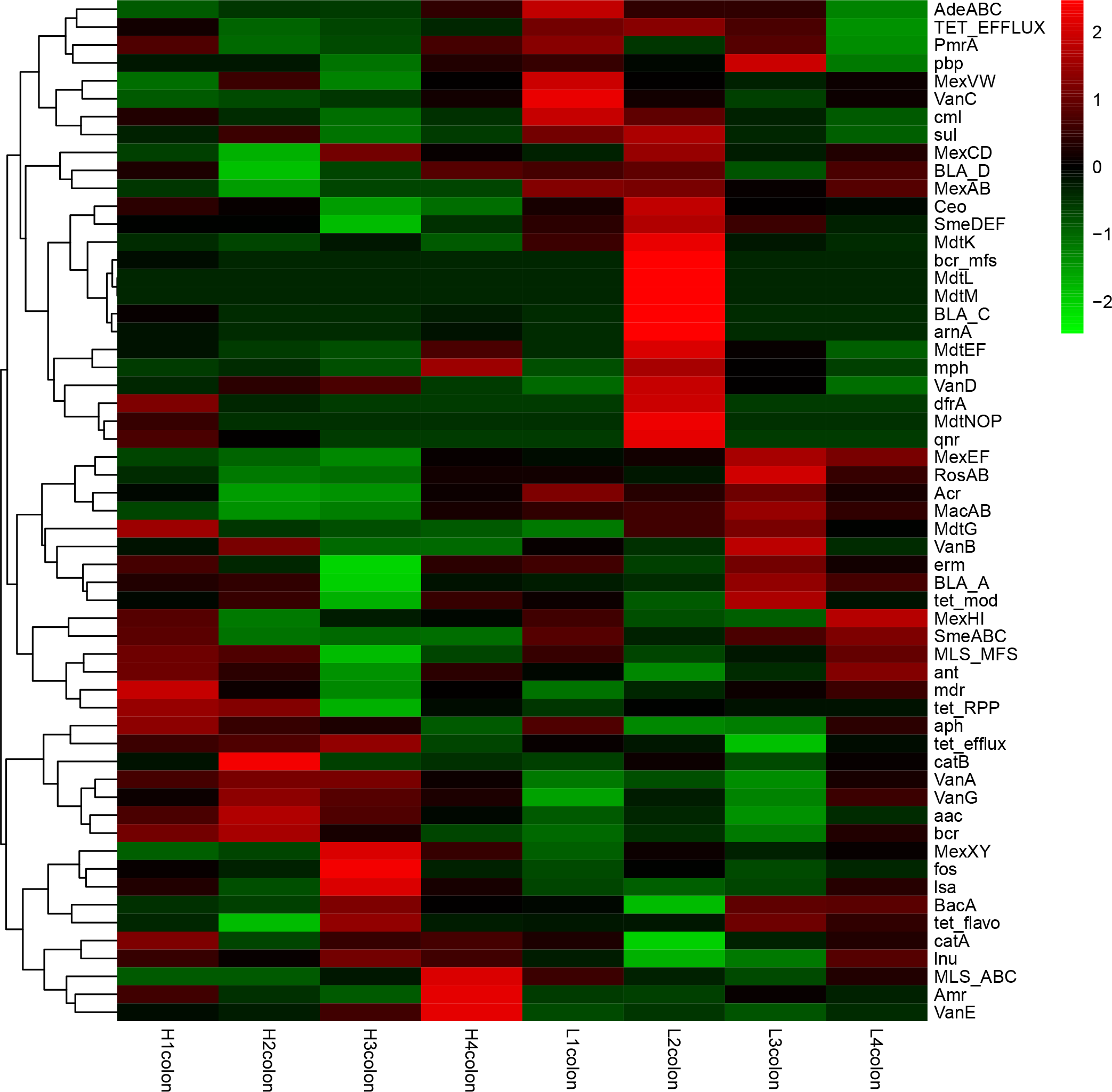

Supplement: Supplementary Figure 6 — Heatmap diagram showing differences in the abundances of antibiotic resistant genes in the colonic microbiota between the Hco (high FE) and Lco (low FE) groups. FE, feed efficiency. Homogenization control of rows is by z-score. Rows represent the types of antibiotic-resistant genes, and the columns represent the samples. [file Image_6.TIF]

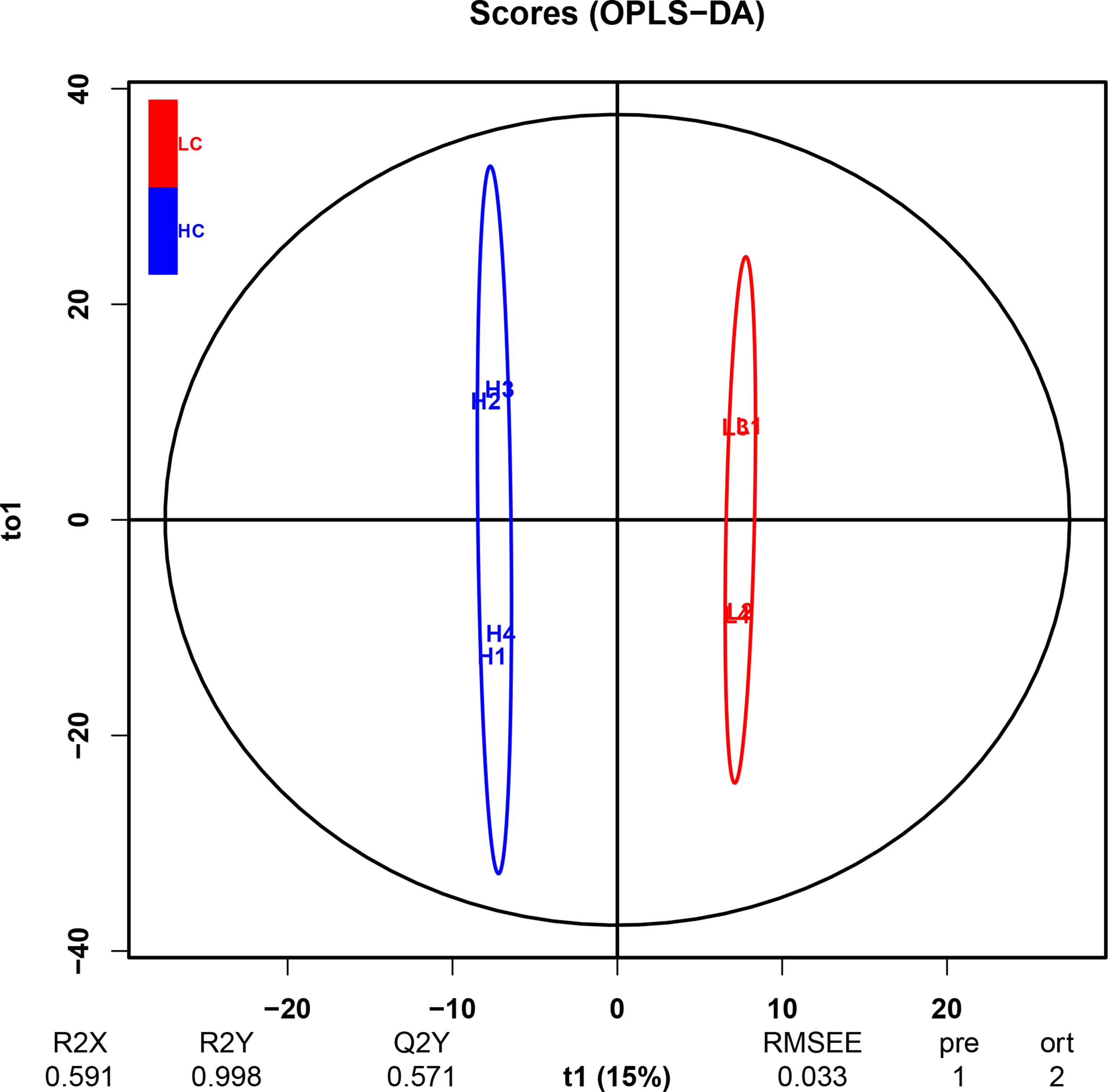

Supplement: Supplementary Figure 7 — OPLS-DA of the microbial metabolites in pigs with high and low feed conversion ratios (FCRs). OPLS-DA, orthogonal projections to latent structures- discriminant analysis. R2X and R2Y represent the interpretation rate of the built model to the X and Y matrices, respectively, where the X matrix is the metabolite quantitative matrix, the Y matrix is the sample-grouping matrix, and Q2 is the predictive ability if the model can distinguish the correct sample grouping by metabolic expression. [file Image_7.TIF]
