# Supplementary material for: Variations in Microbial Diversity and Metabolite Profiles of Female Landrace Finishing Pigs With Distinct Feed Efficiency
Source: Front Vet Sci. 2021 Jul 9;8:702931. doi: 10.3389/fvets.2021.702931 (PMC8299115; doi:10.3389/fvets.2021.702931)
Supplement: Supplementary Table 1 — Feed conversion ratios (FCRs) for fecal samples of selected individuals. DFI, daily feed intake; ADG, average daily gain over the assessed feeding period; BW, body weight. [file Table_1.DOCX]

Supplementary Table 1 Feed conversion ratios of fecal samples of selected individuals.

| Group | HFCR | LFCR | P-value |
| --- | --- | --- | --- |
| n | 20 | 20 |  |
| FCR | 2.049±0.065 | 2.834±0.145 | 2.522E-23 |
| DFI | 1.883±0.124 | 2.245±0.214 | 1.047E-07 |
| ADG | 0.920±0.074 | 0.794±0.087 | 1.582E-05 |
| Initial BW | 50.860±1.444 | 50.535±0.528 | 0.350 |
| Finial BW | 92.274±3.237 | 86.266±4.170 | 1.002E-05 |

DFI - daily feed intake. ADG - average daily gain over the assessed feeding period. BW - body weight.
