# Supplementary material for: Variations in Microbial Diversity and Metabolite Profiles of Female Landrace Finishing Pigs With Distinct Feed Efficiency
Source: Front Vet Sci. 2021 Jul 9;8:702931. doi: 10.3389/fvets.2021.702931 (PMC8299115; doi:10.3389/fvets.2021.702931)
Supplement: Supplementary Table 2 — Individuals selected for metagenomics analysis. Half-siblings, H1co and L1co, H2co and L2co; Full-siblings, H3co and L3co, H4co and L4co. [file Table_2.DOCX]

**Supplementary Table 2 Individuals selected for metagenomics.**

| Group | ID | Sample | Initial BW (kg) | Final BW (kg) | FCR | Father ID | Mother ID |
| --- | --- | --- | --- | --- | --- | --- | --- |
| Hco | 143014 | H1colon | 49.4 | 92.5 | 2.02 | LLDA27813008791 | LLNHPF713043113 |
|  | 143106 | H2colon | 50.5 | 93.9 | 1.99 | LLNHPF711011101 | LLNHPF712131314 |
|  | 126606 | H3colon | 50.2 | 92.5 | 2.03 | LLDA27813008791 | LLNHPF712304013 |
|  | 130504 | H4colon | 51.5 | 92 | 2.09 | LLNHPF712246400 | LLNHPF714116103 |
| Lco | 124110 | L1colon | 50.6 | 81.8 | 3.12 | LLDA27813008791 | LLNHPF714105513 |
|  | 144013 | L2colon | 50.1 | 86.2 | 2.8 | LLNHPF711011101 | LLNHPF713012411 |
|  | 126614 | L3colon | 51.5 | 86.8 | 2.67 | LLDA27813008791 | LLNHPF712304013 |
|  | 130506 | L4colon | 49.7 | 84.3 | 2.79 | LLNHPF712246400 | LLNHPF714116103 |

Half-sib: H1co and L1co, H2co and L2co; Full-sib: H3co and L3co, H4co and L4co.
