# Supplementary material for: Variations in Microbial Diversity and Metabolite Profiles of Female Landrace Finishing Pigs With Distinct Feed Efficiency
Source: Front Vet Sci. 2021 Jul 9;8:702931. doi: 10.3389/fvets.2021.702931 (PMC8299115; doi:10.3389/fvets.2021.702931)
Supplement: Supplementary Table 3 — 16S rRNA gene amplicon sequencing of fecal microbes from pigs with high and low feed efficiency (FE). [file Table_3.DOCX]

**Supplementary Table 3 16S rRNA gene amplicon sequencing of fecal microbes with high and low feed efficiency.**

| Sample | Raw PE | Raw Tags | Clean Tags | Effective Tags | Base(nt) | Avglen(nt) | Q20% | Q30% | GC% | Effective% |
| --- | --- | --- | --- | --- | --- | --- | --- | --- | --- | --- |
| H1 | 65747 | 51920 | 40927 | 38268 | 15838242 | 414 | 97.5 | 94.85 | 53 | 58.2 |
| H2 | 60912 | 48631 | 38605 | 36837 | 15297488 | 415 | 97.56 | 94.94 | 53.12 | 60.48 |
| H3 | 54004 | 43623 | 34389 | 32545 | 13487813 | 414 | 97.52 | 94.89 | 53.02 | 60.26 |
| H4 | 52457 | 42465 | 34630 | 32673 | 13497508 | 413 | 97.55 | 94.94 | 53.29 | 62.29 |
| H5 | 66256 | 53070 | 42853 | 39568 | 16352614 | 413 | 97.47 | 94.78 | 53.13 | 59.72 |
| H6 | 62104 | 49823 | 39564 | 37005 | 15340496 | 415 | 97.45 | 94.75 | 53.09 | 59.59 |
| H7 | 60616 | 48513 | 38914 | 35995 | 14864158 | 413 | 97.51 | 94.87 | 53.21 | 59.38 |
| H8 | 55566 | 44943 | 36470 | 32952 | 13553256 | 411 | 97.54 | 94.93 | 53.45 | 59.3 |
| H9 | 69666 | 56058 | 45200 | 42146 | 17400363 | 413 | 97.52 | 94.89 | 53.28 | 60.5 |
| H10 | 56274 | 45624 | 36162 | 34054 | 14133362 | 415 | 97.48 | 94.79 | 53.22 | 60.51 |
| H11 | 59569 | 48086 | 38690 | 35897 | 14832993 | 413 | 97.48 | 94.84 | 53.04 | 60.26 |
| H12 | 62779 | 50297 | 40455 | 37765 | 15602163 | 413 | 97.46 | 94.75 | 53.29 | 60.16 |
| H13 | 54311 | 42993 | 33932 | 32352 | 13440202 | 415 | 97.43 | 94.65 | 53.55 | 59.57 |
| H14 | 50892 | 41155 | 33153 | 30234 | 12528596 | 414 | 97.53 | 94.95 | 52.98 | 59.41 |
| H15 | 61787 | 49384 | 38874 | 36323 | 15103698 | 416 | 97.51 | 94.86 | 52.85 | 58.79 |
| H16 | 50708 | 40183 | 31260 | 29624 | 12322851 | 416 | 97.42 | 94.63 | 52.84 | 58.42 |
| H17 | 57801 | 45345 | 35278 | 32084 | 13350250 | 416 | 97.51 | 94.9 | 52.67 | 55.51 |
| H18 | 58719 | 46496 | 36879 | 34690 | 14356272 | 414 | 97.53 | 94.94 | 52.99 | 59.08 |
| H19 | 52363 | 42289 | 33241 | 31366 | 13079406 | 417 | 97.57 | 94.99 | 52.84 | 59.9 |
| H20 | 53263 | 42902 | 34160 | 32062 | 13233500 | 413 | 97.56 | 95 | 52.99 | 60.2 |
| L1 | 66460 | 53014 | 42157 | 39429 | 16361292 | 415 | 97.44 | 94.67 | 53.34 | 59.33 |
| L2 | 57554 | 45458 | 35752 | 33448 | 13862423 | 414 | 97.42 | 94.65 | 53.27 | 58.12 |
| L3 | 59069 | 46958 | 37205 | 35139 | 14577820 | 415 | 97.43 | 94.64 | 53.44 | 59.49 |
| L4 | 67173 | 52834 | 41870 | 38755 | 16026137 | 414 | 97.51 | 94.89 | 52.96 | 57.69 |
| L5 | 53956 | 42273 | 33314 | 31699 | 13113765 | 414 | 97.5 | 94.86 | 53.07 | 58.75 |
| L6 | 43531 | 35177 | 29099 | 26158 | 10744948 | 411 | 97.64 | 95.17 | 53.12 | 60.09 |
| L7 | 63778 | 50601 | 40361 | 38418 | 15940849 | 415 | 97.51 | 94.84 | 53.21 | 60.24 |
| L8 | 50673 | 40166 | 31945 | 29639 | 12221301 | 412 | 97.5 | 94.86 | 53.1 | 58.49 |
| L9 | 51696 | 41157 | 32224 | 28864 | 12009720 | 416 | 97.46 | 94.76 | 53.31 | 55.83 |
| L10 | 60039 | 47552 | 37607 | 35357 | 14650398 | 414 | 97.47 | 94.79 | 53.25 | 58.89 |
| L11 | 67631 | 55196 | 46260 | 42234 | 17319876 | 410 | 97.65 | 95.18 | 53.17 | 62.45 |
| L12 | 55844 | 45157 | 36957 | 34735 | 14293392 | 411 | 97.55 | 94.93 | 53.5 | 62.2 |
| L13 | 64915 | 50809 | 40060 | 38246 | 15846472 | 414 | 97.44 | 94.74 | 53.03 | 58.92 |
| L14 | 56575 | 44802 | 35506 | 33184 | 13812948 | 416 | 97.47 | 94.75 | 53.06 | 58.65 |
| L15 | 61581 | 49814 | 40720 | 37255 | 15349242 | 412 | 97.53 | 94.92 | 53.26 | 60.5 |
| L16 | 66383 | 54270 | 44222 | 42714 | 17611685 | 412 | 97.59 | 95.03 | 53.4 | 64.34 |
| L17 | 57847 | 46286 | 37125 | 33887 | 13928392 | 411 | 97.52 | 94.94 | 52.84 | 58.58 |
| L18 | 54681 | 43453 | 34679 | 30750 | 12689868 | 413 | 97.52 | 94.92 | 53.07 | 56.24 |
| L19 | 62779 | 49934 | 39296 | 36796 | 15246843 | 414 | 97.48 | 94.81 | 53.11 | 58.61 |
| L20 | 53299 | 43225 | 35615 | 32733 | 13457175 | 411 | 97.61 | 95.11 | 52.99 | 61.41 |
