# Supplementary material for: Variations in Microbial Diversity and Metabolite Profiles of Female Landrace Finishing Pigs With Distinct Feed Efficiency
Source: Front Vet Sci. 2021 Jul 9;8:702931. doi: 10.3389/fvets.2021.702931 (PMC8299115; doi:10.3389/fvets.2021.702931)
Supplement: Supplementary Table 4 — Alpha diversity indices of fecal microbes in pigs with high and low feed efficiency (FE). n = 20 in each measurement. Shannon and Simpson indices were used to assess biodiversity. PD whole tree index was based on the phylogenetic tree. Chao1 indices were used to estimate the number of OTUs and microbial richness. The observed species index shows the number of OTUs actually observed. [file Table_4.DOCX]

**Supplementary Table Alpha diversity indexes of fecal microbes in high and low feed efficiency.**

| Group | Simpson | Shannon | PD_whole_tree | Chao1 | Observed_species |
| --- | --- | --- | --- | --- | --- |
| HFCR | 0.979±0.015 | 7.806±0.492 | 113.702±8.499 | 3593.595±460.644 | 1724.5±172.461 |
| LFCR | 0.987±0.007 | 8.102±0.27 | 121.883±11.952 | 4283.941±860.685 | 1921.9±262.764 |
| P_value | 0.036 | 0.024 | 0.017 | 0.003 | 0.008 |

n = 20 in each measurement; Shannon and Simpson indices were used to assess biodiversity; PD whole tree index was based on the phylogenetic tree; Chao1 indices were used to estimate the number of OTUs and microbial richness; the observed species index shows the number of OTUs actually observed.
