# Supplementary material for: Variations in Microbial Diversity and Metabolite Profiles of Female Landrace Finishing Pigs With Distinct Feed Efficiency
Source: Front Vet Sci. 2021 Jul 9;8:702931. doi: 10.3389/fvets.2021.702931 (PMC8299115; doi:10.3389/fvets.2021.702931)
Supplement: Supplementary Table 5 — Summary of metagenomic sequencing data. [file Table_5.DOCX]

**Supplementary Table 5 Summary of metagenomic sequencing data.**

| Sample | Group | Assembly Length(bp) | Number of contigs | N50(bp) | N90(bp) | Max(bp) | Average Size(bp) |
| --- | --- | --- | --- | --- | --- | --- | --- |
| H1colon | Hco | 160,380,236 | 145,934 | 1,158 | 566 | 103,633 | 1,098 |
| H2colon | Hco | 160,339,410 | 140,521 | 1,223 | 572 | 132,391 | 1,141 |
| H3colon | Hco | 153,725,170 | 143,119 | 1,128 | 567 | 120,276 | 1,074 |
| H4colon | Hco | 163,590,931 | 143,365 | 1,253 | 574 | 102,419 | 1,141 |
| L1colon | Lco | 175,896,703 | 168,765 | 1,081 | 563 | 41,448 | 1,042 |
| L2colon | Lco | 197,876,312 | 183,095 | 1,143 | 568 | 114,161 | 1,080 |
| L3colon | Lco | 146,811,064 | 136,905 | 1,125 | 565 | 53,892 | 1,072 |
| L4colon | Lco | 162,201,257 | 146,547 | 1,178 | 569 | 129,838 | 1,106 |
